# Childhood and contemporaneous inflammation in depersonalisation and derealisation: Longitudinal evidence from the Avon Longitudinal Study of Parents and Children

**DOI:** 10.1016/j.bbih.2026.101273

**Published:** 2026-05-24

**Authors:** Evelyn Dilkes, Helge Gillmeister, Katie Daughters

**Affiliations:** a Institute of Social and Economic Research, University of Essex, Essex, UK; b Department of Psychology, University of Essex, Essex, UK

**Keywords:** Depersonalisation, Derealisation, Interleukin-6, C-Reactive protein, Inflammation, Anxiety, Dissociation

## Abstract

**Background:**

Depersonalisation (DP) and derealisation (DR) are dissociative experiences that disrupt an individual's perception of themself and their environment. Recent evidence suggests a potential role for inflammatory processes. This study examined longitudinal associations between interleukin-6 (IL-6), C-reactive protein (CRP), and DP and DR across development.

**Methods:**

Data were drawn from the Avon Longitudinal Study of Parents and Children. IL-6 was measured at age 9 (n = 2606) and CRP at ages 9, 15, and 24 (n = 3323). DP and DR symptoms were assessed at ages 12, 17, and 24. Generalised linear mixed models examined associations between inflammatory markers and DP and DR using both early-life and time-varying approaches.

**Results:**

IL-6 at age 9 was not associated with DP or DR at any age. Higher CRP at age 9 was associated with increased odds of DR at age 12 but reduced odds of DR at ages 17 and 24. Higher CRP at age 9 was also associated with reduced odds of DP at age 24. Time-varying analyses differed: higher CRP at 24 was associated with reduced odds of DR at 24, but CRP measured in mid-adolescence was not associated with DR at 17, highlighting differences between fixed childhood and time-varying inflammatory models.

**Conclusions:**

Inflammatory processes may relate differently to DP and DR across development. Childhood CRP showed associations with later DP and DR, whereas contemporaneous CRP was associated only with DR, suggesting differing developmental patterns. These findings may reflect immune adaptation or cognitive mechanisms involved in the maintenance of symptoms.

## 1 Introduction

Depersonalisation and derealisation (DPDR) are dissociative symptoms characterised by alterations in self-perception and perception of the external world. Depersonalisation (DP) involves feelings of detachment from one's thoughts, emotions, body, or actions, often accompanied by perceptual distortions, a disrupted sense of time, emotional numbing, and a diminished sense of self (American Psychiatric Association, 2013). Derealisation (DR), in contrast, refers to an altered perception of the external environment, where surroundings may appear unreal, dreamlike, or visually distorted (American Psychiatric Association, 2013).

These symptoms can be adaptive and frequently manifest in response to traumatic stress but can become persistent and therefore dysfunctional (Simeon, 2004). If symptoms do not remit, individuals may be diagnosed with Derealisation-Depersonalisation Disorder (DDD), which is considered uncommon (approximately 1-2% of the population may qualify for a diagnosis; Hunter et al., 2004; Yang et al., 2023), However, up to 74% of people may experience DPDR at some point in their lives (Hunter et al., 2004), thus these diagnostic rates likely underestimate the true scale of DPDR.

Attaining a diagnosis of DDD can take between 7 and 12 years (Baker et al., 2003; Michal et al., 2016). This delay is compounded by lacking awareness among healthcare providers, leaving individuals with DDD vulnerable to misdiagnosis or unproductive medical consultations (Medford et al., 2005). Therefore, there is an increased likelihood that many individuals with DPDR remain undiagnosed. The use of large, population-based studies that include measurement of DPDR provide an opportunity for accurate epidemiological estimates of DPDR, as well as reliable models of DPDR trajectories. This is particularly important within DPDR research, as the symptoms are often chronic (Baker et al., 2003). Biological mechanisms involved in DPDR is an essential area of research, as DDD currently lacks a recommended biological treatment target, with traditional pharmacological intervention demonstrating inconsistent and limited efficacy (Wang et al., 2023).

DDD has been associated with dysregulation of the limbic system (Phillips et al., 2001; Sierra and Berrios, 1998), autonomic nervous system (Millman et al., 2024), and hypothalamic-pituitary-adrenal (HPA) axis (Simeon et al., 2001, 2007). More recently, research has begun to investigate the role of inflammation in dissociative symptoms broadly, though investigations within DPDR remain limited. Elevated inflammatory markers, such as C-reactive protein (CRP), interleukin-6 (IL-6), and tumour necrosis factor-alpha (TNF-α), are demonstrated as associated with dissociative symptoms (Bizik et al., 2011; Powers et al., 2019). Additionally, individuals with the dissociative-PTSD subtype – characterised by high levels of DPDR – exhibit higher CRP compared to those with non-dissociative PTSD, suggestive of a link between CRP and DPDR (Jarkas et al., 2021). Further, broader theoretical and empirical work has proposed interactions between bodily signalling, immune and neural processes, and self-related cognition and body ownership (e.g. (Barnsley et al., 2011; Ciaunica et al., 2023; Damasio, 2003).

IL-6 and CRP are of particular interest in DPDR given shared antecedents of chronic stress. For example, lack of home ownership or low parental education, low social position and self-reported childhood trauma, are associated with increased concentrations of IL-6 and CRP (Carpenter et al., 2010; Lin et al., 2016; Miller and Chen, 2007; Packard et al., 2011), suggesting they are markers of biological embedding of early adversity (Berens et al., 2017). Similarly, childhood adversity is associated with dissociative symptoms (King et al., 2020; Quiñones, 2022; Rafiq et al., 2018; Thomson and Jaque, 2018; Vonderlin et al., 2018). Therefore, CRP and IL-6 are likely candidates to be associated with dissociative symptoms.

Only one study has investigated inflammation and DPDR specifically, identifying dysregulated inflammatory markers, including reduced CRP and complement C1q subcomponent B, alongside increased alpha-1-antichymotrypsin in people with DDD (Zheng et al., 2024). Although based on only a small clinical sample, this study does point to potential chronic low-grade inflammation in people with DPDR. However, evidence of the role of IL-6 is mixed: one study found increased levels of IL-6 were strongly correlated with somatoform dissociation (involving disturbances or alterations in motor and sensory functioning, perception, or bodily experience, but without a known medical cause), but not with a broader measure of dissociation (Bob et al., 2010), in a sample of 40 inpatients with unipolar depression.

Despite growing insights into inflammatory mechanisms underlying DPDR and dissociation, research remains limited by small, clinical samples. Studies that focus only on diagnosed patients restrict statistical power and limit generalisability to the broader population that experiences sub-clinical or undiagnosed DPDR. The present study addresses these limitations by investigating the relationship between inflammatory markers and DPDR symptoms in a large, prospective general population cohort, thereby providing novel insights into potential biological correlates of DDD. Importantly, anxiety and depressive symptoms have been linked to both inflammatory processes (Renna et al., 2018; Mac Giollabhui et al., 2021) and DPDR (Černis et al., 2025; Michal et al., 2024) and are therefore included in the present study as confounding variables.

To the best of our knowledge, the only UK dataset to hold data on both DPDR and inflammatory markers is the Avon Longitudinal Study of Parents and Children (ALSPAC), containing over three decades of data from children born in the early 1990s. Previous research has effectively utilised the ALSPAC dataset to investigate the impact of IL-6 and CRP on depression (Khandaker et al., 2014; Osimo et al., 2020); psychosis (Donnelly et al., 2022; Perry et al., 2019, 2021) and anxiety (Mongan et al., 2023). Therefore, the ALSPAC dataset is a reliable source of inflammation data that is well poised for testing the longitudinal associations between inflammatory markers and DP and DR.

### 1.1 Study aims

This study examined whether elevated IL-6 and CRP levels were associated with later symptoms of DP and DR. IL-6 and CRP, measured at age 9, were used to assess long-term associations with DP and DR at ages 12, 17, and 24. Additionally, as CRP was additionally measured at ages 15, and 24, further analysis of contemporaneous associations between CRP and DP and DR was conducted. Further, DP and DR were analysed separately in line with evidence suggesting distinct neurobiological underpinnings (Murphy, 2023).

All analyses controlled for a set of covariates to improve model specificity and to minimise confounding effects. Covariates included demographic and socioeconomic (sex, ethnicity, social position), and physical and psychosocial covariates (body mass index [BMI], cumulative ACE scores, and childhood anxiety and depression symptoms).

### 1.2 Hypotheses

H1 Higher IL-6 levels at age 9 will be associated with increased odds of experiencing DP symptoms at ages 12, 17, and 24.

H2 Higher IL-6 levels at age 9 will be associated with increased odds of experiencing DR symptoms at ages 12, 17, and 24.

H3 Higher CRP levels at age 9 will be associated with increased odds of experiencing DP symptoms at ages 12, 17, and 24.

H4 Higher CRP levels at age 9 will be associated with increased odds of experiencing DR symptoms at ages 12, 17, and 24.

H5 Higher CRP levels at each developmental stage (ages 9, 15, and 24) will be associated with increased odds of experiencing DP symptoms at 12, 17, and 24.

H6 Higher CRP levels at each developmental stage (ages 9, 15, and 24) will be associated with increased odds of experiencing DR symptoms at 12, 17, and 24.

## 2 Methods and materials

### 2.1 Cohort Description and sample sizes

**Fig. 1:**
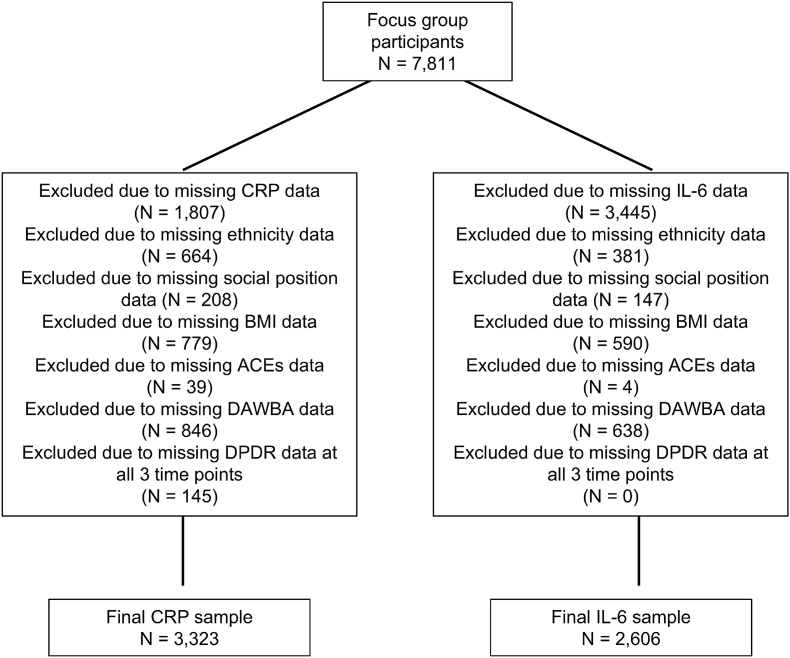
Participant diagram illustrating exclusions due to missing data and final analytic samples for CRP and IL-6 analyses.

Data were drawn from ALSPAC, a large, population-based birth cohort from the United Kingdom. Pregnant women residing in Avon with expected delivery dates between April 1991 and December 1992 were invited to participate (Boyd et al., 2013; Fraser et al., 2013). The analytic sample initially comprised 7811 participants who were invited to, and completed, in-depth focus group interviews. Participants were excluded sequentially due to missing biomarker, covariate, or outcome data (Fig. 1). This resulted in final analytic samples of 3323 participants for the CRP analyses and 2606 participants for the IL-6 analyses. Ethical approval was obtained from the ALSPAC Ethics and Law Committee, and written informed consent was provided by all participants and their parents. Participant characteristics are provided in Table 1.

**Table 1:** Participant characteristics at baseline (age 12).

Demographic	CRP Dataset	IL-6 Dataset
	N (%)	
**Total**	3323 (100)	2606 (100)

**Sex**		
Male	1592 (48)	1295 (50)
Female	1731 (52)	1311 (50)

**Ethnicity**		
White	3206 (97)	2511 (97)
Non-white	117 (3)	95 (3)

**Registrar General's Social Class Scale – Parents' employment**		
I (Professional	437 (13)	338 (13)
II (Managerial/Technical)	1203 (36)	956 (37)
III – N (Skilled non-manual occupations)	452 (14)	370 (14)
III – M (Skilled manual occupations)	901 (27)	682 (26)
IV – Partly-skilled occupations	263 (8)	209 (8)
V – Unskilled occupations	67 (2)	51 (2)

**Cumulative ACEs**		
Mean (SD)	0.84 (1.20)	0.85 (1.19)

**Body-mass Index**		
Mean (SD)	19.79 (3.49)	19.80 (3.48)

**Childhood Anxiety**^a^		
0.5%	1426 (42.91)	1142 (43.82)
3%	1681 (50.59)	1294 (49.65)
∼15%	160 (4.82)	124 (4.76)
∼50%	30 (0.9)	25 (0.96)
>70%	26 (0.78)	21 (0.81)

**Childhood Depression**^a^		
<0.01%	2047 (61.6)	1596 (61.24)
∼0.5%	1182 (35.57)	936 (35.92)
∼15%	73 (2.2)	57 (2.19)
∼50%	20 (0.6)	16 (0.61)
∼70%	1 (0.03)	1 (0.04)

a Childhood anxiety and depression were derived from the Development and Well-Being Assessment (DAWBA). Categories represent computer-generated probability bands indicating the likelihood that the child meets diagnostic criteria for any anxiety disorder or depressive disorder according to DSM-IV and ICD-10 algorithms.

### 2.2 Inflammatory marker assessment

At age 9 (Focus@9), non-fasting blood samples were collected and assayed in 2008 after approximately 7.5 years of storage with no freeze–thaw cycles. Interleukin-6 (IL-6) was measured using an enzyme-linked immunosorbent assay (ELISA; R&D Systems Europe Ltd), and high-sensitivity C-reactive protein (hsCRP) was measured using an automated particle-enhanced immunoturbidimetric assay (Roche Diagnostics GmbH). All assay coefficients of variation were <5%.

At age 15 (Teen Focus 3), fasting blood samples were processed and stored at −80 °C and assayed within 12 months at the University of Glasgow (Professor Naveed Sattar's laboratory) using the same high-sensitivity hsCRP particle-enhanced immunoturbidimetric method. At age 24 (Focus@24+), hsCRP was assayed using the Cardiac C-Reactive Protein (Latex) High Sensitive kit (Roche Diagnostics GmbH).

Participants reporting a recent infection within the past two weeks were excluded. IL-6 levels at age 9 ranged from 0.1 to 13.9 pg/mL hsCRP levels ranged from 0.1 to 67.44 mg/L at age 9, 0.7 to 72.55 mg/L at age 15, and 0.1 to 14.93 mg/L at age 24. Individuals with hsCRP concentrations >10 mg/L were excluded at all waves to minimise the influence of acute inflammatory responses. IL-6 and hsCRP values were log-transformed prior to analysis to account for skewed distributions.

### 2.3 Depersonalisation and derealisation assessment

DP and DR were assessed at ages 12, 17 and 24. To measure DP, individuals were asked if they had “ever felt that they were not a real person, not part of the living world”. To measure DR, individuals were asked if they had “ever felt that the world was unreal, that things around them were like a stage set”. Participants responded on a 3-point scale (never, sometimes, or frequently). Due to low frequencies in the higher symptom categories, responses were dichotomised: participants reporting ‘frequently’ or 'sometimes' experiencing DP or DR were coded as 1 (experienced), while those who had never experienced these symptoms were coded as 0, creating a dichotomous variable for each DP and DR. This approach was used to avoid sparse cell counts and ensure sufficient statistical power and model stability (see also DeCoster et al., 2009). To assess whether this dichotomisation of DP and DR influenced results, sensitivity analyses were conducted using a more restrictive outcome definition in which only “frequently” responses were classified as cases (see Supplementary Table 2–5).

**Fig. 2:**
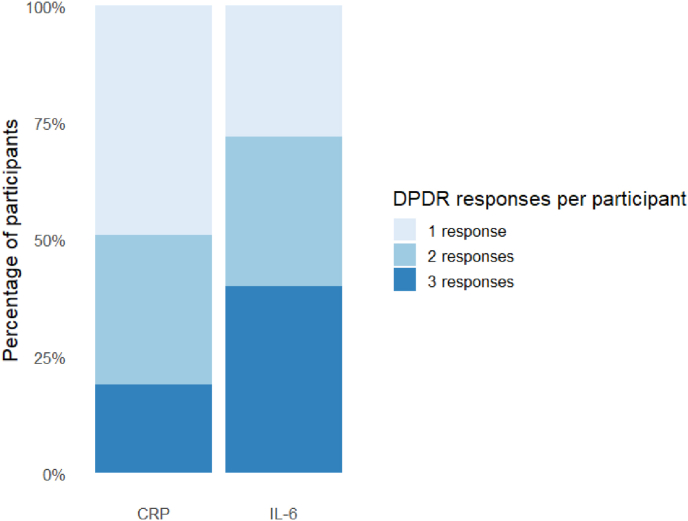
Number of DPDR responses per participant across waves.

**Fig. 3:**
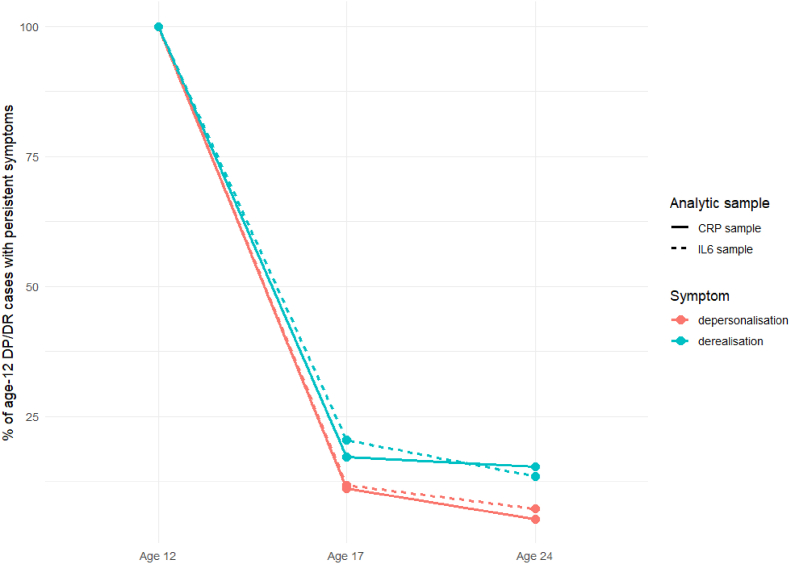
Persistence of depersonalisation and derealisation symptoms from ages 12 to 24.

At age 12, 8% reported DP and 5% reported DR, decreasing to approximately 3–4% at ages 17 and 24 across both the CRP and IL-6 analytic samples (Table 2). Most participants contributed DPDR data at multiple waves, with a higher proportion of participants in the IL-6 sample providing responses at all three time points compared with the CRP sample (Fig. 2). Across both analytic samples, DR showed greater persistence from ages 12 to 17 and 24 than DP (Fig. 3).

**Table 2:** Depersonalisation and derealisation prevalence across time.

	CRP N (%)				IL6 N (%)			
	DP		DR		DP		DR	
	No	Yes	No	Yes	No	Yes	No	Yes
Age 12	2320 (92)	194 (8)	2380 (95)	134 (5)	2326 (92)	198 (8)	2387 (95)	137 (5)
Age 17	1475 (97)	51 (3)	1463 (96)	63 (4)	1692 (97)	54 (3)	1671 (96)	75 (4)
Age 24	1677 (97)	48 (3)	1649 (96)	77 (4)	1336 (97)	36 (3)	1299 (95)	73 (5)

### 2.4 Confounding variables

Several potential confounders measured in childhood or at baseline DP and DR measurement were included as covariates. Ethnicity was classified as white or non-white based on maternal reports of both parents during pregnancy. Sex was recorded at birth as male or female. Social position was determined by paternal occupation during pregnancy using the UK Registrar General's Social Class system; maternal social position was substituted if available and paternal data were missing. BMI was calculated (kg/mˆ2) at age 12. Cumulative childhood adversity was derived using ALSPAC's longitudinally constructed adversity variables. As these measures are pre-defined within the ALSPAC dataset, the indicators included in the cumulative score reflect those available in the ALSPAC-derived variables. Cumulative adversity was calculated by summing binary indicators of exposure across two developmental periods (ages 0–5 and 6–11 years). Indicators included physical abuse, emotional abuse, exposure to domestic violence, bullying, and sexual abuse at ages 0–5, and the same indicators plus emotional neglect at ages 6–11. Each indicator was coded as present or absent. Finally, childhood anxiety and depression were included as potential confounders given their known associations with both inflammatory markers and DP and DR. To minimise the risk of overadjustment, only baseline anxious or depressive psychopathology measured at 7 years were included as fixed confounders. Such psychopathology were measured using DAWBA probability bands indicating the likelihood of meeting DSM-IV or ICD-10 criteria for any anxiety or depressive disorder. All confounders variables were treated as fixed and were included only in adjusted models.

### 2.5 Missing data

Attrition analyses were conducted to examine whether participants included in the analytic sample differed from those excluded due to missing data. Baseline demographic, socioeconomic, and mental health characteristics were compared between groups using chi-squared tests for categorical variables and independent samples t-tests for continuous variables. Results are presented in Supplementary Table 1.

## 3 Data analysis

This study was preregistered on AsPredicted (registration number 142136). In line with ALSPAC governance protocols, raw data cannot be publicly shared; access is granted through a formal application process (https://www.bristol.ac.uk/alspac/). Data were analysed in RStudio (v3.3.4) using the packages dplyr (Wickham, 2015), glmmTMB (Brooks et al., 2017), and ggplot2 (Wickham, 2016).

Generalised linear mixed models (GLMMs) with a binomial distribution and logit link were used to account for the repeated-measures structure of the data and the binary nature of the DPDR outcomes. Random intercepts were included for participant identifiers. IL-6 (age 9) and hsCRP (ages 9, 15, and 24) were the primary predictors. Adjusted models additionally included sex, ethnicity, social position, BMI, cumulative ACE scores, and childhood anxiety and depression as confounders. Separate datasets were used for IL-6 and hsCRP analyses to maximise statistical power due to differences in biomarker availability (see Cohort Description).

Associations between inflammatory markers and DPDR were examined using two complementary analytic approaches. First, IL-6 and CRP measurements at age 9 were modelled longitudinally, examining whether childhood inflammation was associated with long-term DP or DR at ages 12, 17 and 24.

Second, to examine whether associations between CRP and DP or DR varied across development, time-varying CRP measured contemporaneously with DP and DR assessment was modelled across waves (hsCRP measured at ages 9, 15, and 24 in relation to DPDR assessed at ages 12, 17, and 24).

IL-6 and hsCRP scores were standardised (z-scored) prior to analysis. Odds ratios therefore represent the change in odds associated with a one standard deviation increase in symptom severity. To account for multiple testing, p-values for the primary analyses were adjusted using the Benjamini–Hochberg procedure to control the false discovery rate at 5%. Full model specifications and diagnostics are reported in the Supplementary Materials (Model Specification and Model Diagnostics).

## 4 Results

### 4.1 Interleukin-6 at age 9

A one standard deviation (SD) increase in IL-6 at age 9 was not significantly associated with the odds of experiencing DP at age 12 (OR = 1.21, 95% CI: 0.83–1.75, p = .330), age 17 (OR = 1.25, 95% CI: 0.69–2.28, p = .463), or age 24 (OR = 1.76, 95% CI: 0.91–3.40, p = .095). These associations remained non-significant after adjustment for covariates (age 12: aOR = 1.19, 95% CI: 0.82–1.73, p = .368; age 17: aOR = 1.25, 95% CI: 0.69–2.27, p = .463; age 24: aOR = 1.75, 95% CI: 0.91–3.37, p = .096). Full model statistics are presented in Table 3.

**Table 3:** Odds ratios for associations between inflammatory markers measured at different ages and depersonalisation and derealisation symptoms at 12, 17 and 24.

Predictor (Age measured)	Outcome (Age measured)	Unadjusted OR (95% CI)	p	Adjusted aOR (95% CI)	p
IL-6 (9)	DP (12)	1.21 (0.83–1.75)	0.330	1.19 (0.82–1.73)	0.368
	DP (17)	1.25 (0.69–2.28)	0.463	1.25 (0.69–2.27)	0.463
	DP (24)	1.76 (0.91–3.40)	0.095	1.75 (0.91–3.37)	0.096
	DR (12)	1.02 (0.69–1.49)	0.934	1.00 (0.68–1.47)	0.981
	DR (17)	1.15 (0.73–1.82)	0.539	1.15 (0.73–1.82)	0.539
	DR (24)	1.08 (0.66–1.76)	0.766	1.07 (0.66–1.74)	0.770
---	---	---	---	---	---
hsCRP (9)	DP (12)	1.03 (0.67–1.57)	0.891	1.02 (0.66–1.57)	0.930
	DP (17)	0.46 (0.22–0.97)	0.041	0.46 (0.22–0.97)	0.042
	DP (24)	0.35 (0.16–0.74)	**.007∗∗**	0.35 (0.16–0.75)	**.007∗∗**
	DR (12)	1.56 (1.03–2.37)	**.037∗**	1.54 (1.01–2.36)	0.056
	DR (17)	0.27 (0.13–0.56)	**<.001∗∗∗**	0.27 (0.13–0.56)	**<.001∗∗∗**
	DR (24)	0.19 (0.10–0.38)	**<.001∗∗∗**	0.20 (0.10–0.38)	**<.001∗∗∗**
---	---	---	---	---	---
hsCRP (9)	DP (12)	1.31 (0.89–1.93)	0.172	1.32 (0.89–1.96)	0.166
hsCRP (15)	DP (17)	0.77 (0.33–1.75)	0.527	0.76 (0.33–1.74)	0.519
hsCRP (24)	DP (24)	0.75 (0.37–1.51)	0.419	0.75 (0.37–1.51)	0.415
hsCRP (9)	DR (12)	2.28 (1.52–3.41)	**<.001∗∗∗**	2.31 (1.53–3.47)	**<.001∗∗∗**
hsCRP (15)	DR (17)	0.49 (0.23–1.03)	0.060	0.48 (0.23–1.02)	0.059
hsCRP (24)	DR (24)	0.34 (0.19–0.63)	**<.001∗∗∗**	0.34 (0.19–0.63)	**<.001∗∗∗**

**Note:** OR = Odds Ratio; aOR = Adjusted Odds Ratio; CI = Confidence Interval. Adjusted models control for sex, ethnicity, social position, BMI, cumulative ACE score, childhood anxiety, and childhood depression, none of which were independent predictors of DP or DR in any model. Full model parameters, including all covariate statistics, are provided in Supplementary Table S6–S11. Significant values are bolded (∗∗∗p < .001, ∗∗p < .01, ∗p < .05).

Similarly, a one SD increase in IL-6 was not associated with the odds of experiencing DR at age 12 (OR = 1.02, 95% CI: 0.69–1.49, p = .934), age 17 (OR = 1.15, 95% CI: 0.73–1.82, p = .539), or age 24 (OR = 1.08, 95% CI: 0.66–1.76, p = .766). Adjusted models produced comparable results (age 12: aOR = 1.00, 95% CI: 0.68–1.47, p = .981; age 17: aOR = 1.15, 95% CI: 0.73–1.82, p = .539; age 24: aOR = 1.07, 95% CI: 0.66–1.74, p = .770). Full model statistics are presented in Table 3.

### 4.2 C-reactive protein at age 9

For DP, a one SD increase in hsCRP at age 9 was not associated with the odds of experiencing DP at age 12 (OR = 1.03, 95% CI: 0.67–1.57, p = .891). However, a one SD increase in hsCRP was associated with reduced odds of experiencing DP at age 17 (OR = 0.46, 95% CI: 0.22–0.97, p = .041) and age 24 (OR = 0.35, 95% CI: 0.16–0.74, p = .007). These associations remained after adjustment for covariates (age 17: aOR = 0.46, 95% CI: 0.22–0.97, p = .042; age 24: aOR = 0.35, 95% CI: 0.16–0.75, p = .007), while the association at age 12 remained non-significant (aOR = 1.02, 95% CI: 0.66–1.57, p = .930). Full model statistics are presented in Table 3.

For DR, a one SD increase in hsCRP was associated with increased odds of experiencing DR at age 12 (OR = 1.56, 95% CI: 1.03–2.37, p = .037), but decreased odds at age 17 (OR = 0.27, 95% CI: 0.13–0.56, p < .001) and age 24 (OR = 0.19, 95% CI: 0.10–0.38, p < .001). Following adjustment, the age-12 association was attenuated and no longer statistically significant (aOR = 1.54, 95% CI: 1.01–2.36, p = .056), while reduced odds remained significant at age 17 (aOR = 0.27, 95% CI: 0.13–0.56, p < .001) and age 24 (aOR = 0.20, 95% CI: 0.10–0.38, p < .001). Full model statistics are presented in Table 3.

### 4.3 Time-varying CRP at ages 9, 15 and 24

For DP, a one SD increase in hsCRP at age 9 was not associated with the odds of experiencing DP at age 12 (OR = 1.31, 95% CI: 0.89–1.93, p = .172). A one SD increase in hsCRP at age 15 was not associated with the odds of experiencing DP at age 17 (OR = 0.77, 95% CI: 0.33–1.75, p = .527). A one SD increase in hsCRP at age 24 was not associated with the odds of experiencing DP at age 24 (OR = 0.75, 95% CI: 0.37–1.51, p = .419). These associations remained non-significant after adjustment (age 12: aOR = 1.32, 95% CI: 0.89–1.96, p = .166; age 17: aOR = 0.76, 95% CI: 0.33–1.74, p = .519; age 24: aOR = 0.75, 95% CI: 0.37–1.51, p = .415). Full model statistics are presented in Table 3.

For DR, a one SD increase in hsCRP at age 9 was associated with increased odds of experiencing DR at age 12 (OR = 2.28, 95% CI: 1.52–3.41, p < .001), while a one SD increase in hsCRP at age 24 was associated with decreased odds of experiencing DR at age 24 (OR = 0.34, 95% CI: 0.19–0.63, p < .001). There was no association between a one SD in increase in hsCRP at age 15 and the odds of experiencing DR at age 17 (OR = 0.49, 95% CI: 0.23–1.03, p = .060). Adjusted models demonstrated similar patterns, with significant associations for age 12 (aOR = 2.31, 95% CI: 1.53–3.47, p < .001) and age 24 (aOR = 0.34, 95% CI: 0.19–0.63, p < .001), while the age-17 association remained non-significant (aOR = 0.48, 95% CI: 0.23–1.02, p = .059). Full model statistics are presented in Table 3.

## 5 Discussion

This study examined the longitudinal relationship between childhood IL-6, CRP, and DP and DR, as well as the contemporaneous relationship between CRP and DP and DR in the ALSPAC cohort. To our knowledge, this is the first study to investigate the long-term associations between IL-6 and CRP on the odds of experiencing DP and DR. Our findings contribute to the growing body of literature suggesting that immune system dysregulation may be associated with dissociative symptoms broadly (Bizik et al., 2011; Bob et al., 2010; Jarkas et al., 2021; Powers et al., 2019), as well as DPDR specifically (Zheng et al., 2024).

Contrary to expectations, IL-6 measured at age 9 was not significantly associated with the odds of experiencing DP or DR at age 12, 17 or 24 in unadjusted and adjusted models. Therefore, H1, H2 were not supported. These findings contrast with previous research linking childhood IL-6 to psychopathology; for example, IL-6 at age 9 and depression at age 19 (Chu et al., 2019) and depressive and psychotic symptoms at age 24 (Perry et al., 2021) within the ALSPAC. Thus, childhood inflammation might relate differently to long-term DP and DR compared with other psychiatric outcomes.

The current study extends previous mixed findings between IL-6 and dissociative symptoms (Bob et al., 2010). While previous research highlighted a link between IL-6 and somatoform dissociation, but not a broader dissociation measure, the current study identifies that childhood IL-6 is not associated with DP or DR across development. This was surprising, given the overlap between somatoform dissociation and DP and DR but may reflect time of IL-6 measurement and characteristics of the samples (Bob et al. used inpatients with unipolar depression, mean age 42.3 ± 6.8), as well as correlational analyses that did not control for depressive symptoms.

A further explanation for the lack of association in the current study may be due to the sample size following non-complete-case filtering. Sensitivity analyses utilising the maximum available sample (unadjusted for complete covariate data) indicated a significant association between IL-6 and DP at age 24 (see Supplementary Table 12). This suggests that statistical power may have influenced the primary analyses. Further research using larger cohorts with more complete covariate data may help clarify whether childhood IL-6 independently predicts DP later in life.

Additionally, the present findings suggested that CRP may relate differently to DP and DR over time. While it was hypothesised that increased CRP at age 9 would predict increased odds of DPDR later in life, our findings instead suggested that increased CRP at age 9 predicted a 65% lower odds of experiencing DP at age 24 per one SD increase in CRP, as well as a 73% and 80% lower odds of experiencing DR at age 17 and 24, respectively.

A notable finding was the discrepancy between CRP at age 9 and time-varying CRP models for DP and DR. While increased CRP measured at age 9 was associated with reduced odds of DP at age 24, CRP measured contemporaneously with the DP was not associated with DP at any age. In contrast, time-varying CRP exhibited a divergent, age-dependent relationship with DR. Increases in CRP at age 9 was associated with higher odds of experiencing DR at age 12, whereas higher CRP at age 24 was associated with lower odds of experiencing DR at age 24. Interestingly, CRP measured at age 15 was not significantly associated with the odds of experiencing DR at age 17, despite CRP at age 9 being associated with lower odds of DR at that age.

These patterns suggest an important role for elevated CRP in childhood, indicating that early inflammatory activity may be associated with reduced odds of experiencing both DP and DR later in life. Evidence from the contemporaneous analyses further suggests that, at least for DR, the relationship between CRP and symptoms may shift across development. In early adolescence, higher CRP was associated with increased odds of DR, consistent with typical expectations regarding inflammation and psychological symptoms. However, this pattern appeared to reverse over time, such that higher CRP in adulthood was associated with reduced odds of DR.

To the authors’ knowledge, only one previous study has specifically investigated inflammation in DP and DR. In a clinical sample of 30 individuals with depersonalisation–derealisation disorder (mean age ≈24), individuals with DDD showed lower CRP levels compared with healthy controls (Zheng et al., 2024). Supporting our findings, reduced CRP was specifically linked to increased emotional numbing, a feature commonly associated with DP. However, the present findings suggest a broader developmental pattern, whereby CRP demonstrates age-dependent associations across both DP and DR.

The present longitudinal analyses therefore reveal inflammatory patterns that cross-sectional studies cannot detect. Specifically, the relationship between inflammation and DP and DR appears to vary across development, with early CRP elevations associated with increased odds of DR during early adolescence, but higher CRP levels associated with reduced symptom odds in adulthood.

These findings may reflect physiological adaptation within inflammatory systems. Chronic stress is highly associated with DP and DR, and has been proposed to produce immune habituation or exhaustion over time (Barthel et al., 2025; Dhabhar, 2014), resulting in lower inflammatory activity despite the persistence of psychological symptoms. Within this framework, early inflammatory responses may be associated with the emergence of DR, whereas later stages may reflect a weakening of the relationship between inflammatory activity and DP and DR as physiological systems adapt to chronic stress.

This interpretation aligns with cognitive–behavioural models of DDD (Hunter et al., 2014), which posit that DP and DR may initially arise in response to physiological stress but are subsequently maintained through psychological mechanisms such as threat monitoring and maladaptive interpretations of perceptual changes. According to this model, these psychological mechanisms tend to be a downstream consequence of DP and DR, characterised by obsessive self-monitoring of symptoms specifically, rather than a habitual trait which may be captured by childhood anxious or depressive psychopathology that was controlled for in the present study.

The finding that CRP demonstrated stronger age-dependent associations with DR than DP supports the possibility that these dimensions reflect partially distinct biological processes. DR has been linked to disruptions in sensory integration and temporal–parietal processing (Murphy, 2023), neural systems involved in integrating multisensory information to construct coherent perceptions of the external environment. These systems have also been proposed to interact with inflammatory signalling pathways that can alter neural connectivity and perceptual processing (Schmitz et al., 2025). Within this framework, inflammatory activity may more readily influence neural circuits involved in environmental perception than those underpinning self-related processing, potentially contributing to derealisation experiences. These findings align with broader accounts proposing that immune and neural processes jointly contribute to self-related cognition and embodied experience (e.g. (Barnsley et al., 2011; Ciaunica et al., 2023; Damasio, 2003). Given that dissociative symptoms reflect disturbances in the normal integration of self-related information, future research integrating inflammatory markers with neural and behavioural data may therefore help clarify how early immune activity shapes the developmental trajectory of such symptoms.

Beyond absolute levels of inflammatory markers, growing attention has turned to the concept of biological instability, for example allostatic load (AL): defined as “the cost of chronic exposure to a fluctuating or heightened neural or neuroendocrine response resulting from repeated or chronic environmental challenge” (McEwen and Stellar, 1993).

Considering AL is critical given its established association with psychopathology, including increased risk of depression, anxiety, and suicidality (Gou et al., 2025). While DP and DR are traditionally conceptualized as dissociative phenomena, they may also represent prolonged physiological stress responses, making them particularly relevant to an AL perspective. The present findings suggest that CRP dysregulation, a key feature of allostatic overload, may be related to long-term DP and DR. Particularly, the robust longitudinal-yet-reversing association between CRP and DR across time may reflect immune habituation subsequent to AL, and CRP at age 9 being associated with DP and DR across time could suggest biological embedding of stress, evident through dysregulated CRP.

In further support of a role for AL, individuals with DPDR frequently report chronic stress histories, such as ACEs (King et al., 2020; Quiñones, 2022; Thomson and Jaque, 2018; Vonderlin et al., 2018), and dissociative disorders linked to maltreatment are associated with smaller amygdala and hippocampal volumes (Vermetten et al., 2006), regions closely tied to chronic stress and AL (Danese and McEwen, 2012), as well as anxiety (Davis, 1992) and PTSD (Shin et al., 2006). Thus, the co-occurrence of DPDR and immune dysregulation supports the view that DPDR may represent a manifestation of allostatic overload (McEwen, 2004).

Importantly, stress may become chronic not only due to external adversity but also through internalization – where DPDR itself functions as a persistent stressor (Hunter et al., 2003), perpetuating physiological dysregulation and reinforcing the cycle of allostatic strain. In the present study cumulative adversity did not attenuate the relationship between CRP and DPDR, nor was it independently associated with DPDR, but previous research demonstrating a relationship between ACEs and DPDR does suggest that it may be ACE specific, for example, emotional abuse is linked to DPDR as the strongest predictor of symptoms (Quiñones, 2022, Simeon et al., 2001b; King et al., 2020), and therefore future research should use detailed assessments of ACEs, rather than count methods which may lack the granularity to detect the association between specific ACE types and DP and DR.

### 5.1 Strengths and limitations

The present study offers several key strengths. First, use of large, population-based longitudinal datasets (IL-6 = 2606; CRP = 3323) enabled robust tracking of inflammatory markers and DP and DR across developmental stages, allowing for the largest longitudinal study of DP and DR, while including key biological mechanisms that further this essential field of research. Further, this population provided a more representative view of DPDR than smaller clinical studies, which is crucial given that most cases of DDD go undiagnosed for up to 12 years (Baker et al., 2003; Michal et al., 2016).

Second, although DP and DR are frequently classified as a single phenomenon, they are phenomenologically distinct: DP involves alterations in bodily self-experience, while DR reflects changes in the perception of the external world, despite sharing the core feature of a subjective detachment from reality. Despite their overlap in clinical presentation and shared diagnostic category, the present findings support their disaggregation, given that they may have different trajectories dependent on childhood versus contemporaneous inflammatory assessment. Recognizing these distinctions is important to advance understanding of DPDR, and may help inform more targeted research and clinical strategies for diagnosis, prognosis, and treatment.

Finally, the inclusion of a wide range of covariates, including demographic, psychosocial, and psychological factors, represents an improvement over previous research, wherein it may have not been methodologically feasible to control for several confounders (for example through correlations or analyses with small sample sizes). This comprehensive adjustment strengthens confidence that the observed associations are less likely artifacts of co-occurring psychopathology or individual characteristics.

This study also has several limitations. First, IL-6 was measured only at age 9 years, limiting insights into the association between IL-6 and DPDR measured contemporaneously. Further, the dichotomisation of DP and DR does not capture symptom severity or frequency, constraining our ability to assess dose-response relationships or subthreshold variation. Further, while associations were identified, this study cannot determine causality; it remains unclear if inflammation directly contributes to symptom development.

Finally, the use of complete-case analysis across models resulted in a reduction in the sample size. While this ensured sample consistency, it may have reduced statistical power to detect associations between inflammatory markers and DP and DR, particularly within the IL-6 analysis. Supplementary analyses using a larger available-case sample that had not removed individuals with missing covariate data indicated a potentially strong association between IL-6 and DP at age 24; therefore, it is possible that the present study was underpowered to detect smaller associations between IL-6 and DP within unadjusted and adjusted models. However, these sensitivity analyses should be used only as information for methodological rigour in future studies, emphasising the need for large samples with low attrition for covariates to clarify whether childhood IL-6 is associated with the odds of experiencing DP later in life.

### 5.2 Clinical implications and future directions

Future research should continue to examine inflammatory markers and broader indices of AL in relation to DP and DR. Methodologically, future studies would benefit from detailed and repeated assessments of DP and DR to capture symptom severity and developmental trajectories. Longitudinal cohort studies could integrate brief assessments of DP and DR, such as the 8-item Brief Dissociative Symptoms Scale (Macia et al., 2023). Further, integrative designs incorporating immune, neuroendocrine, and neuroimaging measures may also help clarify how inflammatory processes may be associated with neural systems involved in self-processing and perceptual integration, which can be achieved within cohort studies with biomarker and neuroimaging data should they measure DP and DR. Additionally, given the mutual associations between ACEs, DP and DR, and inflammatory markers, future research should conduct formal mediation analyses to provide evidence of potential pathways that link ACEs to DPDR.

From a clinical perspective, trauma-focused and cognitive–behavioural interventions remain the primary evidence-based treatments for DDD. However, emerging evidence suggests that interventions targeting systemic inflammation – such as physical activity, anti-inflammatory lifestyle modifications (Gaesser et al., 2012), and mindfulness-based stress reduction (O'Toole et al., 2018) – may offer potential complementary approaches, particularly in individuals with elevated inflammatory markers. Further research is needed to determine whether targeting inflammatory pathways can improve treatment outcomes in dissociative disorders.

## 6 Conclusion

This study provides longitudinal evidence that inflammatory processes may relate differently to DP and DR across development. While childhood CRP demonstrated associations with later DP and DR, contemporaneous CRP was associated only with DR, suggesting that early-life and current inflammatory activity may play different roles in DP and DR. These findings highlight the importance of distinguishing between DP and DR when investigating biological mechanisms of DP and DR and suggest that developmental timing of inflammatory activity may be relevant to understanding symptoms.

## Declaration of generative AI and AI-assisted technologies in the writing process

During the preparation of this work the author used ChatGPT (OpenAI) for language editing and improving clarity. The author reviewed and edited the content and takes full responsibility for the publication.

## CRediT authorship contribution statement

**Evelyn Dilkes:** Conceptualization, Data curation, Formal analysis, Funding acquisition, Investigation, Methodology, Project administration, Resources, Writing – original draft. **Helge Gillmeister:** Supervision, Writing – review & editing. **Katie Daughters:** Conceptualization, Writing – review & editing.

## Declaration of competing interest

The authors declare that they have no known competing financial interests or personal relationships that could have appeared to influence the work reported in this paper.

## Data Availability

The authors do not have permission to share data.
