# Supplementary material for: Childhood and contemporaneous inflammation in depersonalisation and derealisation: Longitudinal evidence from the Avon Longitudinal Study of Parents and Children
Source: Brain Behav Immun Health. 2026 May 24;54:101273. doi: 10.1016/j.bbih.2026.101273 (PMC13235410; doi:10.1016/j.bbih.2026.101273)
Supplement: Multimedia component 1 [file mmc1.docx]

**Supplementary Materials**

**Attrition**

Attrition analyses were conducted to examine whether participants included in the analytic sample differed from those excluded due to missing data. Because the analyses used a complete-case approach, participants with missing data on any exposure, covariate, or outcome variable were excluded from the final models.

Categorical baseline variables (sex, ethnicity, parental social position, childhood anxiety, and childhood depression) were compared using Pearson’s chi-squared tests. Continuous variables (body mass index and adverse childhood experiences) were compared using independent samples t-tests. These analyses were conducted to evaluate whether attrition was associated with baseline demographic, socioeconomic, or mental health characteristics.

Attrition analyses indicated that participants included in the CRP analytic sample did not differ significantly from those excluded with respect to sex (χ²(1) = 1.40, p = .237), body mass index (t(6708) = 0.01, p = .991), childhood anxiety (χ²(4) = 4.68, p = .322), or childhood depression (χ²(4) = 1.45, p = .835). Small but statistically significant differences were observed for ethnicity (χ²(1) = 8.03, p = .005), parental social position (χ²(5) = 11.31, p = .046), and adverse childhood experiences (t(7108) = −2.11, p = .035).

Attrition analyses were also conducted for the IL-6 analytic sample. Participants included in the IL-6 analyses differed from those excluded with respect to sex (χ²(1) = 10.63, p = .001) and parental social position (χ²(5) = 14.09, p = .015). However, no significant differences were observed for ethnicity (χ²(1) = 3.59, p = .058), body mass index (t(5840) = 0.25, p = .805), ACE exposure (t(5592) = −1.46, p = .144), childhood anxiety (χ²(4) = 0.70, p = .951), or childhood depression (χ²(4) = 2.24, p = .692).

Overall, these findings suggest that the analytic sample was broadly comparable to the full cohort with respect to most baseline characteristics, although some socioeconomic differences were present.

Supplementary Table 1:

| **Characteristic** | **Included CRP (n = 3323)** | **Excluded CRP** | **p** | **Included IL-6 (n = 2606)** | **Excluded IL-6** | **p** |
| --- | --- | --- | --- | --- | --- | --- |
| **Sex, n (%)** |  |  | .237 |  |  | .**001** |
| Male | 1592 | 2117 |  | 1295 | 2422 |  |
| Female | 1731 | 2428 |  | 1311 | 2860 |  |
| **Ethnicity, n (%)** |  |  | **.005** |  |  | .058 |
| White | 3206 | 3371 |  | 2511 | 4085 |  |
| Non-white | 117 | 175 |  | 95 | 98 |  |
| **Parental social position, n (%)** |  |  | **.046** |  |  | **.015** |
| I (Professional) | 437 | 403 |  | 338 | 509 |  |
| II (Managerial / Technical) | 1203 | 1174 |  | 956 | 1423 |  |
| III – N (Skilled non-manual) | 452 | 424 |  | 370 | 506 |  |
| III – M (Skilled manual) | 901 | 929 |  | 682 | 1156 |  |
| IV (Partly-skilled) | 263 | 315 |  | 209 | 369 |  |
| V (Unskilled) | 67 | 93 |  | 51 | 112 |  |
| **BMI, mean** | 19.82 | 19.82 | .991 | 19.81 | 19.83 | .805 |
| **ACEs (0–11), mean** | 0.86 | 0.80 | **.035** | 0.85 | 0.81 | .144 |
| **Childhood anxiety, n (%)** |  |  | .322 |  |  | .951 |
| 0.5% | 1428 | 1222 |  | 1142 | 1512 |  |
| 3% | 1684 | 1292 |  | 1297 | 1684 |  |
| ~15% | 164 | 127 |  | 127 | 166 |  |
| ~50% | 30 | 20 |  | 25 | 26 |  |
| >70% | 26 | 22 |  | 21 | 27 |  |
| **Childhood depression, n (%)** |  |  | .835 |  |  | .692 |
| <.01% | 2049 | 1595 |  | 1596 | 2039 |  |
| ~0.5% | 1188 | 925 |  | 941 | 1182 |  |
| ~15% | 74 | 59 |  | 58 | 76 |  |
| ~50% | 20 | 12 |  | 16 | 16 |  |
| ~70% | 1 | 0 |  | 1 | 0 |  |

Sensitivity Analysis

To evaluate whether the dichotomisation of the DP and DR outcomes influenced the results, sensitivity analyses were conducted using a more restrictive outcome definition. In the primary analyses, DP and DR symptoms were operationalised as binary variables that included participants reporting symptoms occurring sometimes or more frequently. In the sensitivity analysis, responses indicating sometimes were excluded, such that only participants reporting more frequent symptoms were classified as cases.

Under this more restrictive definition, the number of participants meeting criteria for DPDR symptoms was substantially reduced. Consequently, model estimates became unstable: odds ratios were attenuated, confidence intervals widened considerably, and overall model precision was reduced. These patterns suggest that the models were underpowered when the outcome definition was restricted to frequent DPDR symptoms only.

Given the relatively low prevalence of frequent DP and DR experiences in the cohort, the primary binary outcome definition was retained for the main analyses. This approach allowed the inclusion of participants reporting intermittent DP and DR symptoms while producing more stable and interpretable estimates.

*Supplementary Table 2: Sensitivity analysis examining associations between CRP and depersonalisation symptoms when defining depersonalisation as occurring “frequently”.*

|  | ***OR*** | ***CI*** | ***P*** |
| --- | --- | --- | --- |
| CRP (measured at Age 9) x DP (measured at age 12) | 1.02 | 0.29 – 3.56 | .982 |
| CRP (measured at Age 15) x DP (measured at age 17) | 1.02 | 0.11 – 9.48 | .968 |
| CRP (measured at Age 24) x DP (measured at age 24) | 0.83 | 0.10 – 6.89 | .864 |

Supplementary Table 3: *Sensitivity analysis examining associations between CRP and derealisation symptoms when defining derealisation as occurring “frequently”.*

|  | ***OR*** | ***CI*** | ***P*** |
| --- | --- | --- | --- |
| CRP (measured at Age 9) x DR (measured at age 12) | 0.88 | 0.25-3.10 | .840 |
| CRP (measured at Age 15) x DR (measured at age 17) | 1.29 | 0.17 – 10.07 | .805 |
| CRP (measured at Age 24) x DR (measured at age 24) | 1.32 | 0.15 – 11.47 | .805 |

*Supplementary Table 4: Sensitivity analysis examining associations between IL-6 and depersonalisation symptoms when defining depersonalisation as occurring “frequently”.*

|  | ***OR*** | ***CI*** | ***P*** |
| --- | --- | --- | --- |
| IL-6 (measured at Age 9) x DP (measured at age 12) | 0.89 | 0.09-8.93 | .919 |
| IL-6 (measured at Age 9) x DP (measured at age 17) | 1.10 | 0.06 – 20.29 | .954 |
| IL-6 (measured at Age 9) x DP (measured at age 24) | 0.95 | 0.04 – 20.49 | .976 |

*Supplementary Table 5: Sensitivity analysis examining associations between IL-6 and derealisation symptoms when defining derealisation as occurring “frequently”.*

|  | ***OR*** | ***CI*** | ***P*** |
| --- | --- | --- | --- |
| IL-6 (measured at Age 9) x DR (measured at age 12) | 1.23 | 0.14 – 10.81 | .851 |
| IL-6 (measured at Age 9) x DR (measured at age 17) | 0.98 | 0.06 – 17.29 | .990 |
| IL-6 (measured at Age 9) x DR (measured at age 24) | 0.91 | 0.04 – 19.69 | .951 |

**Full tables**

*Supplementary Table 6: Full unadjusted and adjusted OR for the association between log-transformed IL-6 at age 9 and depersonalisation at ages 12, 17, and 24*

|  | Unadjusted model | | | Adjusted model | | |
| --- | --- | --- | --- | --- | --- | --- |
| **Age** | ***OR*** | ***CI*** | ***P*** | ***aOR*** | ***CI*** | ***P*** |
| IL-6 (measured at Age 9) x DP (measured at age 12) | 1.25 | 0.80-1.94 | .330 | 1.23 | 0.79 - 1.92 | .368 |
| IL-6 (measured at Age 9) x DP (measured at age 17) | 1.30 | 0.64-2.64 | .463 | 1.30 | 0.64 - 2.64 | .463 |
| IL-6 (measured at Age 9) x DP (measured at age 24) | 1.95 | 0.89-4.25 | .095 | 1.94 | 0.89 - 4.21 | .096 |
| Sex |  | | | 1.11 | 0.52 - 2.38 | .789 |
| Ethnicity |  |  |  | 1.68 | 0.30 - 9.36 | .555 |
| Social position |  |  |  | 0.92 | 0.68 - 1.24 | .577 |
| BMI |  |  |  | 0.98 | 0.88 - 1.09 | .747 |
| Cumulative ACE score |  |  |  | 1.13 | 0.85 - 1.49 | .407 |
| Anxiety |  |  |  | 0.87 | 0.48 - 1.59 | .661 |
| Depression |  |  |  | 0.98 | 0.53 - 1.79 | .937 |

**** p < .001, ** p < .01, * p < .05. Significant values are bolded. Unadjusted model refers to the association between log-transformed IL-6 and depersonalisation symptoms with no covariates included. Adjusted model includes sex, ethnicity, social position, BMI, cumulative ACE score, anxiety and depression as covariates.*

*Supplementary Table 7: Full unadjusted and adjusted OR for the association between log-transformed IL-6 at age 9 and derealisation at ages 12, 17, and 24*

|  | Unadjusted model | | | Adjusted model | | |
| --- | --- | --- | --- | --- | --- | --- |
| **Age** | ***OR*** | ***CI*** | ***P*** | ***aOR*** | ***CI*** | ***P*** |
| IL-6 (measured at Age 9) x DR (measured at age 12) | 1.02 | 0.65-1.60 | .934 | 0.99 | 0.63 – 1.57 | .981 |
| IL-6 (measured at Age 9) x DR (measured at age 17) | 1.18 | 0.69-2.04 | .539 | 1.18 | 0.69 – 2.03 | .539 |
| IL-6 (measured at Age 9) x DR (measured at age 24) | 1.09 | 0.61-1.95 | .766 | 1.09 | 0.61 – 1.93 | .770 |
| Sex |  | | | 1.39 | 0.70 – 2.76 | .352 |
| Ethnicity |  |  |  | 1.15 | 0.20 – 6.77 | .876 |
| Social position |  |  |  | 0.86 | 0.65 – 1.13 | .272 |
| BMI |  |  |  | 0.98 | 0.89 – 1.09 | .759 |
| Cumulative ACE score |  |  |  | 1.15 | 0.89 – 1.47 | .289 |
| Childhood anxiety |  |  |  | 1.07 | 0.63 – 1.80 | .800 |
| Childhood depression |  |  |  | 0.87 | 0.49 – 1.54 | .632 |

**** p < .001, ** p < .01, * p < .05. Significant values are bolded. Unadjusted model refers to the association between log-transformed IL-6 and derealisation symptoms with no covariates included. Adjusted model includes sex, ethnicity, social position, BMI, cumulative ACE score, anxiety and depression as covariates.*

Supplementary Table 8: *Full unadjusted and adjusted OR for the association between log-transformed CRP at age 9 and depersonalisation at ages 12, 17, and 24*

|  | Unadjusted model | | | Adjusted model | | |
| --- | --- | --- | --- | --- | --- | --- |
| **Age** | ***OR*** | ***CI*** | ***P*** | ***aOR*** | ***CI*** | ***P*** |
| hsCRP (measured at Age 9) x DP (measured at age 12) | 1.03 | 0.71 - 1.49 | .891 | 1.02 | 0.70-1.48 | .930 |
| hsCRP (measured at Age 9) x DP (measured at age 17) | 0.51 | 0.27 - 0.97 | .041 | 0.51 | 0.27-0.97 | .042 |
| hsCRP (measured at Age 9) x DP (measured at age 24) | 0.40 | 0.20 - 0.77 | **.007** | 0.40 | 0.20-0.77 | **.007** |
| Sex |  | | | 1.20 | 0.51-2.83 | .682 |
| Ethnicity |  |  |  | 1.44 | 0.20-10.61 | .721 |
| Social position |  |  |  | 0.92 | 0.65-1.29 | .617 |
| BMI |  |  |  | 0.96 | 0.84-1.09 | .497 |
| Cumulative ACE score |  |  |  | 1.12 | 0.81-1.53 | .495 |
| Anxiety |  |  |  | 0.87 | 0.45-1.70 | .680 |
| Depression |  |  |  | 1.01 | 0.51-1.97 | .988 |

**** p < .001, ** p < .01, * p < .05. Significant values are bolded. Unadjusted model refers to the association between log-transformed CRP and depersonalisation symptoms with no covariates included. Adjusted model includes sex, ethnicity, social position, BMI, cumulative ACE score, anxiety and depression as covariates.*

*Supplementary Table 9:* *Full unadjusted and adjusted OR for the association between log-transformed CRP at age 9 and derealisation at ages 12, 17, and 24*

|  | Unadjusted model | | | Adjusted model | | |
| --- | --- | --- | --- | --- | --- | --- |
| **Age** | ***OR*** | ***CI*** | ***P*** | ***aOR*** | ***CI*** | ***P*** |
| hsCRP (measured at Age 9) x DR (measured at age 12) | 1.47 | 1.02 – 2.13 | **.037** | 1.44 | 0.99-2.10 | .056 |
| hsCRP (measured at Age 9) x DR (measured at age 17) | 0.32 | 0.17-0.61 | **<.001** | 0.31 | 0.16-0.60 | **<.001** |
| hsCRP (measured at Age 9) x DR (measured at age 24) | 0.24 | 0.13-0.43 | **<.001** | 0.24 | 0.13-0.43 | **<.001** |
| Sex |  | | | 1.45 | 0.61-3.43 | .396 |
| Ethnicity |  |  |  | 0.90 | 0.09-9.00 | .930 |
| Social position |  |  |  | 0.88 | 0.62-1.24 | .458 |
| BMI |  |  |  | 0.96 | 0.84-1.09 | .503 |
| Cumulative ACE score |  |  |  | 1.20 | 0.89-1.62 | .233 |
| Anxiety |  |  |  | 1.09 | 0.58-2.03 | .794 |
| Depression |  |  |  | 0.88 | 0.43-1.77 | .717 |

**** p < .001, ** p < .01, * p < .05. Significant values are bolded. Unadjusted model refers to the association between log-transformed CRP and derealisation symptoms with no covariates included. Adjusted model includes sex, ethnicity, social position, BMI, cumulative ACE score, anxiety and depression as covariates.*

*Supplementary Table 10: Unadjusted and adjusted OR for the association between log-transformed hsCRP at ages 9, 15 and 24 and depersonalisation at ages 12, 17, and 24*

|  | Unadjusted model | | | Adjusted model | | |
| --- | --- | --- | --- | --- | --- | --- |
| **Age** | ***OR*** | ***CI*** | ***P*** | ***aOR*** | ***CI*** | ***P*** |
| hsCRP (measured at Age 9) x DP (measured at age 12) | 1.24 | 0.91 - 1.69 | .172 | 1.25 | 0.91 - 1.71 | .166 |
| hsCRP (measured at Age 15) x DP (measured at age 17) | 0.81 | 0.42 - 1.56 | .527 | 0.80 | 0.41 - 1.56 | .519 |
| hsCRP (measured at Age 24) x DP (measured at age 24) | 0.79 | 0.45 - 1.39 | .419 | 0.79 | 0.45 - 1.39 | .415 |
| Sex |  | | | 0.98 | 0.42 - 2.24 | .952 |
| Ethnicity |  |  |  | 1.30 | 0.17 - 9.73 | .797 |
| Social position |  |  |  | 0.94 | 0.68 - 1.30 | .701 |
| BMI |  |  |  | 0.95 | 0.84 - 1.08 | .424 |
| Cumulative ACE score |  |  |  | 1.09 | 0.80 - 1.49 | .589 |
| Anxiety |  |  |  | 0.88 | 0.46 - 1.70 | .709 |
| Depression |  |  |  | 0.98 | 0.51 - 1.90 | .960 |

**** p < .001, ** p < .01, * p < .05. Significant values are bolded. Unadjusted model refers to the association between log-transformed CRP and depersonalisation symptoms with no covariates included. Adjusted model includes sex, ethnicity, social position, BMI, cumulative ACE score, anxiety and depression as covariates.*

*Supplementary Table 11: Unadjusted and adjusted OR for the association between log-transformed hsCRP at ages 9, 15 and 24 and derealisation at ages 12, 17, and 24*

|  | Unadjusted model | | | Adjusted model | | |
| --- | --- | --- | --- | --- | --- | --- |
| **Age** | ***OR*** | ***CI*** | ***P*** | ***aOR*** | ***CI*** | ***P*** |
| hsCRP (measured at Age 9) x DR (measured at age 12) | 1.93 | 1.40 - 2.66 | **<.001***** | 1.94 | 1.40 - 2.69 | **<.001***** |
| hsCRP (measured at Age 15) x DR (measured at age 17) | 0.57 | 0.31 - 1.02 | .060 | 0.56 | 0.31 - 1.02 | .059 |
| hsCRP (measured at Age 24) x DR (measured at age 24) | 0.42 | 0.26 - 0.69 | **<.001***** | 0.43 | 0.26 - 0.69 | **<.001***** |
| Sex |  | | | 1.11 | 0.49 - 2.50 | .810 |
| Ethnicity |  |  |  | 0.77 | 0.07 - 8.16 | .831 |
| Social position |  |  |  | 0.89 | 0.64 - 1.24 | .503 |
| BMI |  |  |  | 0.95 | 0.84 - 1.07 | .418 |
| Cumulative ACE score |  |  |  | 1.15 | 0.86 - 1.54 | .348 |
| Anxiety |  |  |  | 1.12 | 0.61 - 2.05 | .711 |
| Depression |  |  |  | 1.04 | 0.56 - 1.92 | .908 |

**** p < .001, ** p < .01, * p < .05. Significant values are bolded. Unadjusted model refers to the association between log-transformed CRP and derealisation symptoms with no covariates included. Adjusted model includes sex, ethnicity, social position, BMI, cumulative ACE score, anxiety and depression as covariates.*

**Model specification**

H1–H2: Baseline IL-6 models

$$\text{logit}(P(DP/DR_{it}))=\beta_{0}+\beta_{1}(\text{Time}_{t})+\beta_{2}(\text{IL6}_{i})+\beta_{3}(\text{Time}_{t}\times\text{IL6}_{i})+\beta_{4}(\text{Sex}_{i})+\beta_{5}(\text{Ethnicity}_{i})+\beta_{6}(\text{Social Position}_{i})+\beta_{7}(\text{BMI}_{i})+\beta_{8}(\text{ACEs}_{i})+\beta_{9}(\text{Anxiety}_{i})+\beta_{10}(\text{Depression}_{i})+u_{i}$$

H3–H4: Baseline CRP models

$$\text{logit}(P(DP/DR_{it}))=\beta_{0}+\beta_{1}(\text{Time}_{t})+\beta_{2}(\text{CRP}_{i})+\beta_{3}(\text{Time}_{t}\times\text{CRP}_{i})+\beta_{4}(\text{Sex}_{i})+\beta_{5}(\text{Ethnicity}_{i})+\beta_{6}(\text{Social Position}_{i})+\beta_{7}(\text{BMI}_{i})+\beta_{8}(\text{ACEs}_{i})+\beta_{9}(\text{Anxiety}_{i})+\beta_{10}(\text{Depression}_{i})+u_{i}$$

H5–H6: Time-varying CRP models

$$\text{logit}(P(DP/DR_{it}))=\beta_{0}+\beta_{1}(\text{Time}_{t})+\beta_{2}(\text{CRP}_{it})+\beta_{3}(\text{Time}_{t}\times\text{CRP}_{it})+\beta_{4}(\text{Sex}_{i})+\beta_{5}(\text{Ethnicity}_{i})+\beta_{6}(\text{Social Position}_{i})+\beta_{7}(\text{BMI}_{i})+\beta_{8}(\text{ACEs}_{i})+\beta_{9}(\text{Anxiety}_{i})+\beta_{10}(\text{Depression}_{i})+u_{i}$$

Notation

- $i$= participant
- $t$= time point
- $u_{i}$= random intercept for participant

**Model diagnostics**

All models converged successfully with no optimisation warnings. Multicollinearity among fixed effects was assessed using variance inflation factors, with all values below 3, indicating low collinearity between predictors. Multicollinearity was estimated using the performance package in R. Simulation-based residual diagnostics were conducted using the DHARMa package in R. Bootstrapped outlier tests indicated no influential observations. Dispersion diagnostics suggested underdispersion (dispersion ratios ≈ 0.30–0.40), which is commonly observed in binomial mixed-effects models with repeated observations and does not affect parameter estimation, although it may result in slightly conservative standard errors. Random intercepts were included for participant identifiers to account for repeated observations across waves.

**Available-case analysis**

To assess the potential impact of the complete-case restriction used in the primary analyses, we conducted a sensitivity analysis using all available IL-6 data without filtering participants based on missing covariates. These models therefore represent the association between IL-6 measured at age 9 and DP or DR outcomes when participants were included if outcome data were available, regardless of missingness in other variables.

*Supplementary Table 12: Available-case analysis examining associations between IL-6 measured at age 9 and depersonalisation at ages 12, 17 and 24 (unadjusted models using all available data)*

|  | ***OR*** | ***CI*** | ***P*** |
| --- | --- | --- | --- |
| IL-6 (measured at Age 9) x DP (measured at age 12) | 1.22 | 0.93 – 1.59 | .147 |
| IL-6 (measured at Age 9) x DP (measured at age 17) | 1.28 | 0.82 – 1.99 | .278 |
| IL-6 (measured at Age 9) x DP (measured at age 24) | 2.09 | 1.36 – 3.21 | <.001 |

*Supplementary Table 13: Available-case analysis examining associations between IL-6 measured at age 9 and derealisation at ages 12, 17 and 24 (unadjusted models using all available data)*

|  | ***OR*** | ***CI*** | ***P*** |
| --- | --- | --- | --- |
| IL-6 (measured at Age 9) x DR (measured at age 12) | 1.17 | 0.89 - 1.54 | .254 |
| IL-6 (measured at Age 9) x DR (measured at age 17) | 1.12 | 0.79 - 1.58 | .530 |
| IL-6 (measured at Age 9) x DR (measured at age 24) | 0.90 | 0.63 - 1.31 | .593 |
